# A geographical population analysis of dental trauma in school-children aged 12 and 15 in the city of Curitiba-Brazil

**DOI:** 10.1186/1472-6963-10-203

**Published:** 2010-07-13

**Authors:** Max L Carvalho, Samuel J Moysés, Roberto E Bueno, Silvia Shimakura, Simone T Moysés

**Affiliations:** 1 Pontifical Catholic University of Paraná, Oral Health Postgraduate Programme - Subject Area: Collective Health, Curitiba, PR, Brazil; 2 Polytechnic Centre, Department of Statistics, Federal University of Paraná, Curitiba, PR, Brazil

## Abstract

**Background:**

The study presents a geographical analysis of dental trauma in a population of 12 and 15 year-old school-children, in the city of Curitiba, Brazil (n = 1581), using a database obtained in the period 2005-2006. The main focus is to analyze dental trauma using a geographic information system as a tool for integrating social, environmental and epidemiological data.

**Methods:**

Geostatistical analysis of the database and thematic maps were generated showing the distribution of dental trauma cases according to Curitiba's Health Districts and other variables of interest. Dental trauma spatial variation was assessed using a generalized additive model in order to identify and control the individual risk-factors and thus determine whether spatial variation is constant or not throughout the Health Districts and the place of residence of individuals. In addition, an analysis was made of the coverage of dental trauma cases taking the spatial distribution of Curitiba's primary healthcare centres.

**Results:**

The overall prevalence of dental trauma was 37.1%, with 53.1% in males and 46.7% in females. The spatial analysis confirms the hypothesis that there is significant variation in the occurrence of dental trauma, considering the place of residence in the population studied (Monte Carlo test, p = 0,006). Furthermore, 28.7% of cases had no coverage by the primary healthcare centres.

**Conclusions:**

The effect of the place of residence was highly significant in relation to the response variable. The delimitation of areas, as a basis for case density, enables the qualification of geographical territories where actions can be planned based on priority criteria. Promotion, control and rehabilitation actions, applied in regions of higher prevalence of dental trauma, can be more effective and efficient, thus providing healthcare refinement.

## Background

In recent years the complexity of dental trauma epidemiology has been highlighted in the specialized literature, with successive reports of its increasing prevalence [1,2]. Although it is not consensus that it represents a public health problem, given that its impact on the individual level is often not self-perceived, many are those who advocate that actions to prevent and control this problem in communities cannot be postponed [3-8].

Epidemiologically, there are many geographical and population aspects that can be related to traumatic injuries [9-11]. For example: healthcare service location; access to such services (personal mobility and transport availability); the existence of urban social facilities in the community (schools, community centres and leisure facilities); local infrastructure (basic sanitation, electricity supply, adequate housing and policing); as well as the social support network, given that such social, environmental and health determinants have a direct influence on risk, health promotion and on the prevention and treatment of the diverse injuries to the oral and facial region [12].

Mapped information, using geographical information systems, may allow public health professionals and managers to have a broader vision of the problem and this in turn enables improved decision making and the definition of evidence based public policies that meet the population's needs [13].

Geographic Information Systems (GIS) applied to health sciences have undergone great technological development over time, permitting better characterization and quantification of exposure and its possible determinants, as well as its outcomes [14-18]. Furthermore, sophisticated spatial analysis techniques, integrated with GIS, have brought new perspectives to the field of Epidemiology [3,19-21].

The geographical analysis of the determinants of dental trauma enables promotion, control and rehabilitation actions to be planned as closely as possible to the affected areas, in accordance with the principles of healthcare decentralization adopted in Curitiba, in keeping with the directives of the Brazilian National Health System [22]. We propose to address this problem in Curitiba using a survey of 12 and 15 year-old school-children and treating the occurrence of dental trauma as a binary response. Another objective is to identify the respective coverage of the problem by the Health System in this city.

## Methods

The secondary database used was created from an original field study accepted by a national call for studies and financed by the Brazilian National Council of Technological and Scientific Development (CNPq), having been approved by the Research Ethics Committee of the Pontifical Catholic University of Paraná (CEP-PUCPR No. 528/05). The primary data collection took place in 2005-2006 in the city of Curitiba-PR, Brazil, and the secondary data analysis occurred during 2007-2008.

The primary survey data on dental trauma prevalence were collected in the city's nine Health Districts (*Santa Felicidade*, *Boa Vista*, *Boqueirão*, *Portão*, *Pinheirinho*, *Cajuru*, *Matriz*, *Bairro Novo *and *Cidade Industrial de Curitiba*), and data collection was undertaken together with the application of a questionnaire containing variables considered to be relevant, based on a previous review of the literature.

Sampling design, which aimed to work with World Health Organization (WHO) age groups and indices, included schoolchildren aged 12 and 15, enrolled at public and private schools and resident in the Health Districts. A probabilistic sample was calculated for each District. Expected dental trauma frequency was set at 15% for the population studied in Curitiba, having a 95% confidence interval and power of 95%. The primary sample was comprised of 1581 schoolchildren.

A visual examination was performed with the aid of a wooden spatula. The criterion used for defining and recording dental trauma was that proposed by the British Association for the Study of Community Dentistry (2005), for basic oral health surveys. A pilot study was performed to assess the level of consistency between examiners regarding the dental trauma examination. In order to guarantee the reliability of the records, the examination procedures were standardized beforehand. The details of the database are shown in Table 1, in Results.

**Table 1:** Distribution of dental trauma frequency in 1581 schoolchildren

	Dental trauma n (%)	No dental trauma n (%)	Total n (%)
**Age group**			
12 years	321 (36.48%)	559 (63.52%)	880 (55.66%)
15 years	266 (37.95%)	435 (62.05%)	701 (44.34%)
			
**Sex**			
Male	313 (41.79%)	436 (58.21%)	749 (47.38%)
Female	274 (32.93%)	558 (67.07%)	832 (52.62%)
			
**Type of school**			
Public	392 (39.48%)	601 (60.52%)	993 (62.81%)
Private	195 (33.16%)	393 (66.84%)	588 (37.19%)
			
**Total**	587 (37.13%)	994 (62.87%)	1581 (100%)

Distribution by age, sex, and type of school. City of Curitiba-PR - 2005.

**Figure 1:**
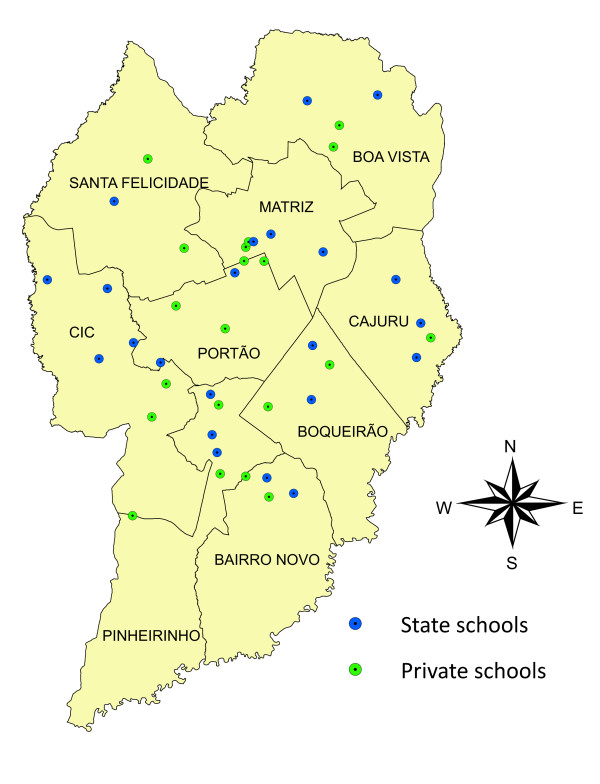
**Georeferencing of the schools analyzed - City of Curitiba, 2005**.

The secondary data analysis was performed on 21 public schools and 20 private schools, georeferenced on the map of the city of Curitiba shown in Figure 1. Data was processed in this study using the records relating to: sex, age, type of school, Health District, presence of trauma, trauma location, cause of trauma and the residential addresses of the sampled individuals. Two further variables were added, grouped by Health District: a) average income of the head of the household (expressed in Brazilian minimum wages); and b) the number of urban social facilities in the Health District, obtained from the Curitiba City website using data provided by the Curitiba Institute of Urban Research and Planning (IPPUC) [23].

The individuals' residential addresses were georeferenced using ARCGIS 9.2 software, although 168 of the 1581 primary records did not contain information capable of being georeferenced. The final number of geocoded points was 1413. The X and Y coordinates of the georeferenced points were added to the initial spreadsheet which was imported in the R statistics program [24], in order to adjust the univariate and multiple regression models and the generalized additive model. Thematic maps of dental trauma distribution, with differentiation between experiencing trauma or not, were generated using ARCGIS 9.2 software.

### Statistical Analyses

Descriptive statistical analyses were initially performed on previously filtered data after performing consistency analysis. Spatial operations were then processed in order to analyze coverage by Curitiba City Health Centres and an estimate was made of trauma case density related to the place of residence, using the Kernel [25] estimation method, whereby the layer generated through the previous procedure was superimposed on average income distribution per Health District.

Regression models were adjusted, analyzing the variables of sex, age, type of school (public or private), average income (expressed in Brazilian minimum wages) of the head of the household and the number of urban social facilities in the District, as independent variables that could exert influence (both of risk and protection) over dental trauma. Initially, place of residence was not taken into consideration. In the study there is a response variable, or outcome, of the binomial type (coded as 1 for individuals with dental trauma experience and 0 for those without dental trauma). This variable is dependent on variables or covariates that may affect the subjects under analysis.

The spatial variation of dental trauma was assessed using a generalized additive model in order to identify and control the independent variables as individual risk-factors and thus determine whether spatial variation is constant or not throughout the region studied, that is to say, whether the additive component of variation in space is significant or not in relation to dental trauma distribution [26].

The study-design justifies the use of a generalized additive model (GAM), which is an extension of the generalized linear models (GLM) with a predictor involving the addition of nonparametric functions or smooth covariate functions [27,28]. An algorithm was used to estimate the optimum bandwidth for the data, as per the method proposed by Kelsall & Diggle (1998), using cross-validation of weighted minimums for the nonparametric regression step. The overall risk test and the identification of areas of low and high dental trauma prevalence, as related to the place of residence of the subjects, were carried out using the Monte Carlo [29] simulation method. By calculating the test's p value, it was possible to evaluate the central hypothesis of the study regarding the existence or not of spatial variation in dental trauma distribution.

### Spatial Operations

Taking the spatial distribution of the City of Curitiba's Health Centres [23], an analysis was made of the coverage of dental trauma cases in this sample. Using the ARCGIS 9.2 software spatial analysis tools, a 1 km radius of coverage was set in an empirical manner, delimiting the greatest potential action of the Health Centres and, following this, a buffer (a polygon associated with an area around the selected object) was created at the points referring to the georeferenced units. Once the cases of dental trauma experience had been distributed, a spatial examination was made of the cases that were not inside the Centres' buffers, that is to say, those that were not inside the potential population coverage area of the Health Centre, and a map was generated of these results.

## Results

### Profile of the Population Studied

Of the 1581 schoolchildren studied in the city of Curitiba, 587 (representing a sample prevalence of 37.1%) had dental trauma experience. Table 1 shows the distribution of dental trauma frequency by age, sex and type of school (public or private).

**Figure 2:**
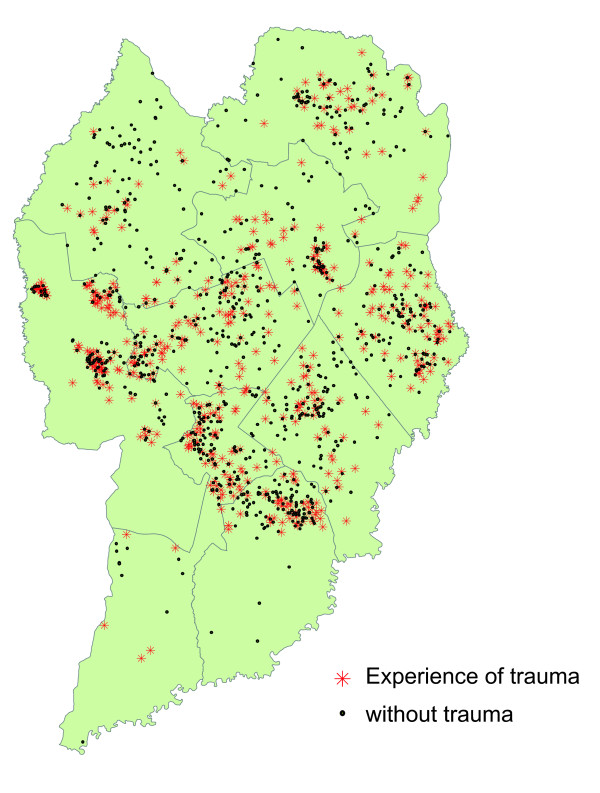
**Georeferencing of the residences of the individuals studied**. City of Curitiba, 2005.

As explained in the Methods section, owing to the lack of information on 168 households, 1413 sample units (residences of the subjects of the study) were georeferenced, and not 1581. Of the 1413 units, 890 had no cases of trauma and 523 had experience of dental trauma as shown in Figure 2. The prevalence of trauma in subjects whose residences were not georeferenced was (64/168 = 38.1%). This prevalence was similar to those whose addresses had been recorded; thus, non-response bias is probably not a problem in this study.

In the original sample, 832 (52.7%) of those studied were male and 749 (47.3%) female. With regard to the age variable, 880 (55.7%) were 12 years old and 701 (44.3%) were 15 years old. The majority of those interviewed stated that they "did not know" the place where the trauma had occurred, followed by "residence" as the most common place of occurrence. As to the cause, 220 (37.5%) stated they "did not know", whilst a "fall" was the second most common cause: 127 (21.6%). Among those who stated that they "did not know", there was 91.4% prevalence of trauma restricted to tooth enamel.

**Figure 3:**
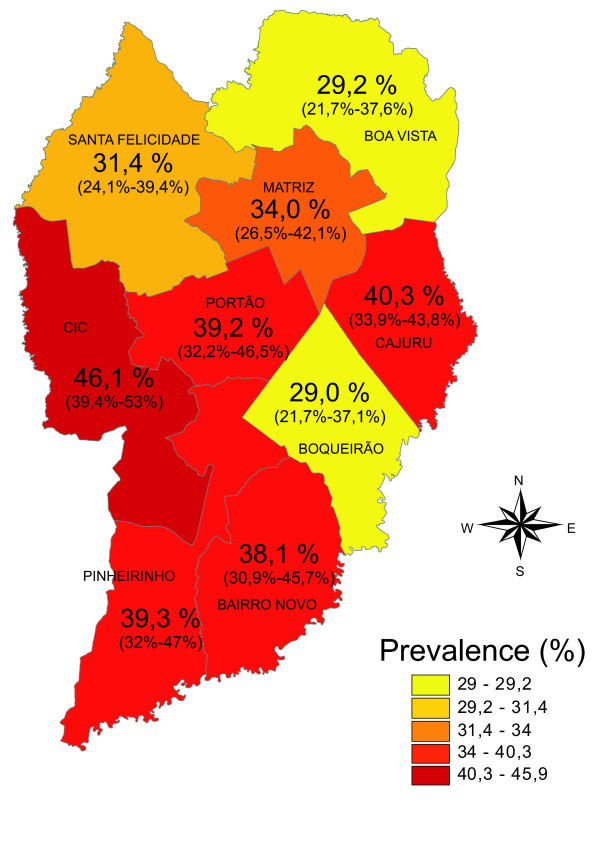
**Dental trauma prevalence per Health District - City of Curitiba, 2005**.

The highest prevalence of dental trauma was found in the *Cidade Industrial de Curitiba *Health District, with 101 cases (46.1%); the *Boqueirão *Health District had the lowest prevalence, with 42 cases (29%), as shown in Figure 3.

With regard to the type of school, the public schools had the largest number of dental trauma, with 392 cases (66.8%), whilst the private schools had 195 cases (33.2%).

### Data Analysis

The estimates for the univariate regression model are shown in Table 2, having the male sex as the base group for the sex variable, public school as the base for the school type variable and 12 as the base for the age variable. The estimates for the multiple regression model with and without the spatial effect are shown in Table 3. Among the covariates studied, the influence of sex, type of school, average income per Health District and the number of urban social facilities in the District was confirmed. Below is the regression model adopted:

**Table 2:** Estimates of the effects of the covariates, univariate logistic regression model, without spatial effect

*Basis/Factor*	*Estimate*	*Standard error*	p-value
Sex	-0.3976	0.1107	**0.0003**
Age	0.0665	0.1109	0.5490
Type of School (public or private)	-0.2615	0.1141	**0.0219**
Average Income	-0.0154	0.0135	0.2531
Number of Urban Facilities	0.0007	0.0005	0.1800

Estimates of the effects of the covariates, using univariate logistic regression model, without spatial effect (95% confidence interval)

**Table 3:** Estimates of the effects of the covariates, multiple logistic regression model

	*Multiple Logistic Regression Adjustment without spatial effect*			*Multiple Logistic Regression Adjustment with spatial effect *		
***Basis/Factor***	***Estimate***	***Standard error***	***p-value***	***Estimate***	***Standard error***	***p-value***

Sex	-0.4132	0.1115	**0.0002**	-0.3953	0.1110	0.0004*
Age	0.0815	0.1124	0.4686	0.1171	0.1120	0.2960
School Type	-0.2565	0.1152	**0.0260**	-0.0996	0.1148	0.3859
Av. Income	-0.0388	0.0161	**0.0158**	-0.0340	0.0159	0.0330*
Number of urban facilities	0.0015	0.0007	**0.0187**	0.0007	0.0007	0.2728

Estimates with and without spatial effect using multiple logistic regression model (95% confidence interval)

$$\begin{array}{l} \log\left\{\frac{p}{1-p}\right\}=\beta_{0}+\beta_{1}*\text{sex}+\beta_{2}\text{*age}+\\ \beta_{3}\text{*schooltype}+\beta_{4}\text{*avincome}+\beta_{5}\text{*facilities} \end{array}$$

In the univariate regression model, sex and school type were found to be significant, having a 95% confidence interval. In the multiple regression without the spatial effect, in addition to the sex and school type variables, the number of urban social facilities and average income per Health District were seen to be significant. After adding the spatial effect to the model, the number of urban social facilities per Health District and the type of school (public or private) were no longer significant in relation to the response variable (p = 0.273), as shown in Table 3.

Using the Monte Carlo simulation method, with 1000 simulations, to test the hypothesis of the existence (or not) of a spatial effect, a value of p = 0.006 was obtained, and therefore the null hypothesis that the spatial effect is constant and equal to zero was discarded.

**Figure 4:**
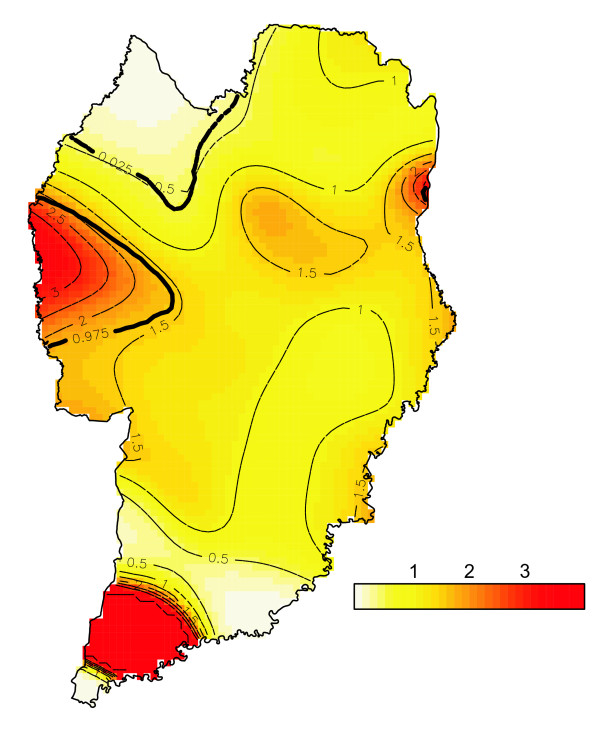
**Estimated risk surfaces, controlled by individual factors**.

The estimated risk surfaces, controlled by individual factors, are presented in Figure 4. Tolerance contours were built to help with the identification of urban areas where prevalence is significantly higher (or lower) than the overall average. The map of the estimated prevalence surfaces shows an area of lower prevalence in the northern region and an area of greater prevalence, related to the place of residence of the subjects in the western region, the latter corresponding mainly to the *Cidade Industrial de Curitiba *Health District. The area in the north has a spatial statistical significance with a 95% confidence interval. A greater prevalence area was also identified in the extreme south, although this area has few georeferenced cases.

**Figure 5:**
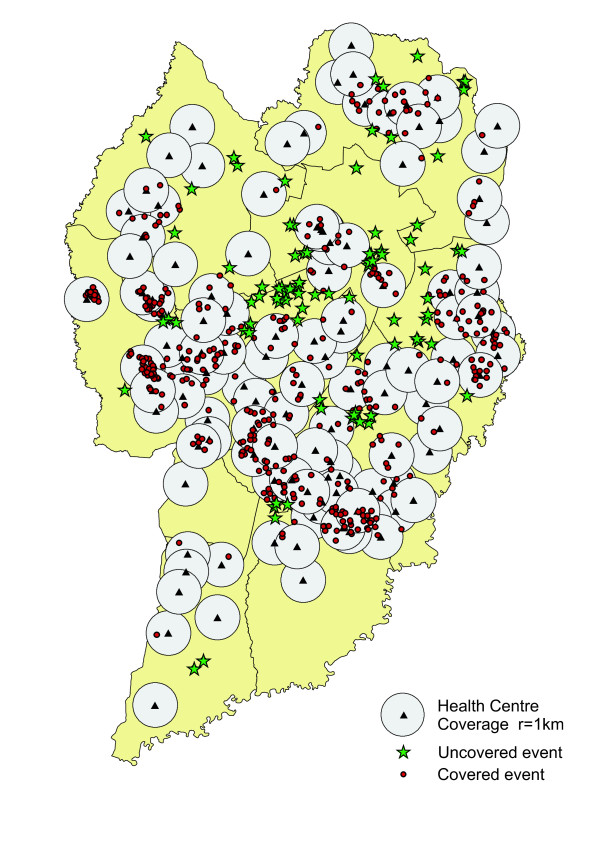
**Relationship between the extent of Health Centre coverage and dental trauma distribution**.

With regard to the issue of the adequacy of the spatial distribution of Curitiba's municipal Health Centres, specifically in relation to the occurrence of dental trauma, a spatial analysis was made of the coverage of the cases studied. Using the methodology described earlier, the map shown in Figure 5 was generated, whereby, according to the analytical model used, 150 of the 523 georeferenced cases of trauma are located outside the Health Centres' coverage area. The points relating to these cases are represented by a star. It can be seen that the coverage areas of several Health Centres overlap and that there is a concentration of Health Centres in the central/west region, where there is a higher density of cases of dental trauma in the population studied.

## Discussion

The spatial variation confirmed by this study leads us to believe that space as a multidimensional attribute may be acting as a proxy for other determinants and variables not collected in the initial survey and which may influence dental trauma prevalence. Other factors have been explored in the literature, such as social capital being a possible explanation of differences in trauma rates, especially in the male sex [4]. The environment, public social policies and social cohesion are determinants relating to geographic areas and have been shown to be factors that influence dental trauma outcomes [3]. Given the possibility of being able to cross-reference processes that give rise to environmental risks, a complementarity of events can be established that enables a global analysis of health risks [15].

Ecological analysis of socio-environmental and epidemiological data may enable, beyond the verification of associations between these phenomena, a better structural understanding of the context in which socio-spatial processes are produced [30]. The analytical model used simulates an approximation to the more widespread socio-structural and environmental conditions that influence the response variable.

An intriguing piece of information observed was that the number of urban social facilities in the Health District and the type of school (public or private) to which each individual had been exposed ceased to have statistical significance in the presence of the spatial effect. It is possible that by including the spatial effect in the model, aggregated ecological determinants may be interacting and having greater influence on the outcome variable, especially in the male sex, so that the District's structural socio-environmental conditions become much more significant and there is a reduction in the intensity of the influence of the individual exposure variables, such as the type of school attended by the schoolchild or the number of urban facilities that he/she can potentially use. As such, an important next step would be the comparison of these regions with data on the families' profiles, especially traumatized individuals of the male sex, based on their material living conditions, in particular in relation to employment and income, and possible gender interactions with the social situation in these regions, including violence and accidents. These findings provide additional elements for future research, in the quest for other contextual determinants and individual variables, including aspects particular to the population being studied, so as to enhance data modelling.

The higher dental trauma prevalence estimated by the thematic map is for the most part contained within the *Cidade Industrial de Curitiba *Health District, in the western region. This region has the second lowest income, suggesting that because of its unfavourable socio-economic conditions this District may have an increased tendency towards dental trauma prevalence in the population.

With regard to the spatial operations performed to analyze the coverage areas of the Health Centres, 28.7% of the cases had no coverage, according to the methodology used. The accessibility of a Health Centre should be an important consideration for service managers, given that it is a fundamental aspect in ensuring that a population has primary healthcare services available, at the least. Clearly, urban planning relating to the spatial location of Health Centres will not be determined by an outcome variable alone, but it is important to observe that various health outcomes often have common causes of risk [31]. Studies, including studies undertaken in Brazil, demonstrate that populations with better socio-economic conditions are the first to be reached by public policies and programs, while the poorest have less coverage and access to health services [32]. This perverse finding, known as the "inverse care law" or the "inverse equality hypothesis" needs to be changed and it is essential that there be equality in the distribution of health resources, and that this be the foremost concern of the governmental sectors [33].

A limitation encountered in the study relates to the address records of the individuals studied, given that in some cases they did not correspond to the official street maps provided by Curitiba City Council (IPPUC), because they are usually in areas of substandard housing and/or illegally occupied areas. On-line address location databases were used (Apontador - http://www.apontador.com.br, Google maps - http://www.maps.google.com.br, Map24 - http://www.br.map24.com) for the points not geocoded by the ARCGIS 9.2 software in order to manually perform the georeferencing of the points cross-referenced in these other databases. Another important limitation of cross-sectional studies is the difficulty of establishing when and where the studied event happened. Furthermore, a large number of those interviewed replied that they "did not know" where the dental trauma had occurred or its cause, thus making impossible a more in-depth analysis of these variables. In more serious cases, it is a problem that can have considerable individual and social impact and as such a new hypothesis can be put forward that in some cases domestic violence and other factors capable of causing embarrassment may have hidden causal links of dental trauma experience. However, in the majority of cases the problem was probably not considered to be serious by those studied, given that these cases referred to tooth enamel fractures.

The question of whether the study results have external validity is often a matter of judgment that depends on the study setting, the characteristics of the participants, the exposures examined, and the outcomes assessed. In this study, in pure statistical terms, the external validity of results was ensured by a probabilistic sample in all Health Districts of Curitiba. The population groups examined are assumed to be relevant for comparisons of urban schoolchildren in most communities in Brazil and elsewhere. Although the average prevalences are somewhat higher than in other studies published in Brazil and internationally, we believe that the methods used in the study can be generalized to other contexts, particularly in results obtained by estimating relative prevalences and the area of dental care coverage of the events.

## Conclusions

The association of individual, environmental and socio-economic data analyzed in a geographical context enables the spatial behaviour of various dimensions of the individual and his/her group to be revealed. The delimitation and posterior qualification of areas, as a basis for case density enable the qualification of geographical territories where actions can be planned based on priority criteria, thus respecting the directives of the Brazilian National Health System.

Promotion, control and rehabilitation actions, applied in regions of greater prevalence and higher density of dental trauma, as demonstrated with regard to dental trauma spatial distribution, can be more effective and efficient, thus providing a greater degree of precision and refinement of healthcare. Spatial analyses can be used as a planning and support tool in the process of organizing the healthcare sector, with the aim of implementing health surveillance systems. In this way a break can be made with the traditional tendency of spontaneous service provision and, in its place, service provision can be planned in keeping with the main illnesses and priority population groups.

In this study, the dental trauma spatial analysis enabled the alternative hypothesis to be accepted, namely that space has an effect on this outcome variable, with significant concentration in Curitiba's western region, with many of the cases identified by places of residence being in areas without the potential coverage of the Health Centres of Curitiba's public health system.

## Competing interests

The authors declare that they have no competing interests.

## Authors' contributions

MLC carried out the review of the literature, wrote part of the article and created the maps by geoprocessing the cases. SJM participated in the study design, the statistical analysis model design, study coordination and helped to write the article. REB participated in the statistical analysis and discussions. SS participated in the study design and performed the statistical analysis. STM carried out the database generation and helped to write the article. All authors read and approved the final text.

## Pre-publication history

The pre-publication history for this paper can be accessed here:

http://www.biomedcentral.com/1472-6963/10/203/prepub
